# A Deep Learning Approach to Denoise Optical Coherence Tomography Images of the Optic Nerve Head

**DOI:** 10.1038/s41598-019-51062-7

**Published:** 2019-10-08

**Authors:** Sripad Krishna Devalla, Giridhar Subramanian, Tan Hung Pham, Xiaofei Wang, Shamira Perera, Tin A. Tun, Tin Aung, Leopold Schmetterer, Alexandre H. Thiéry, Michaël J. A. Girard

**Affiliations:** 1 Ophthalmic Engineering & Innovation Laboratory, Department of Biomedical Engineering, Faculty of Engineering, National University of Singapore, Singapore, Singapore; 2 Singapore Eye Research Institute, Singapore National Eye Centre, Singapore, Singapore; 3 Beijing Advanced Innovation Center for Biomedical Engineering, School of Biological Science and Medical Engineering, Beihang University, Beijing, China; 4 Duke-NUS Graduate Medical School, Singapore, Singapore; 5 Department of Statistics and Applied Probability, National University of Singapore, Singapore, Singapore; 6 Nanyang Technological University, Jurong West, Singapore; 7 Department of Clinical Pharmacology, Medical University of Vienna, Vienna, Austria; 8 Center for Medical Physics and Biomedical Engineering, Medical University of Vienna, Vienna, Austria

**Keywords:** Machine learning, Translational research, Biomedical engineering

## Abstract

Optical coherence tomography (OCT) has become an established clinical routine for the *in vivo* imaging of the optic nerve head (ONH) tissues, that is crucial in the diagnosis and management of various ocular and neuro-ocular pathologies. However, the presence of speckle noise affects the quality of OCT images and its interpretation. Although recent frame-averaging techniques have shown to enhance OCT image quality, they require longer scanning durations, resulting in patient discomfort. Using a custom deep learning network trained with 2,328 ‘clean B-scans’ (multi-frame B-scans; signal averaged), and their corresponding ‘noisy B-scans’ (clean B-scans + Gaussian noise), we were able to successfully denoise 1,552 unseen single-frame (without signal averaging) B-scans. The denoised B-scans were qualitatively similar to their corresponding multi-frame B-scans, with enhanced visibility of the ONH tissues. The mean signal to noise ratio (SNR) increased from 4.02 ± 0.68 dB (single-frame) to 8.14 ± 1.03 dB (denoised). For all the ONH tissues, the mean contrast to noise ratio (CNR) increased from 3.50 ± 0.56 (single-frame) to 7.63 ± 1.81 (denoised). The mean structural similarity index (MSSIM) increased from 0.13 ± 0.02 (single frame) to 0.65 ± 0.03 (denoised) when compared with the corresponding multi-frame B-scans. Our deep learning algorithm can denoise a single-frame OCT B-scan of the ONH in under 20 ms, thus offering a framework to obtain superior quality OCT B-scans with reduced scanning times and minimal patient discomfort.

## Introduction

In recent years, optical coherence tomography (OCT) imaging has become a well-established clinical tool for assessing optic nerve head (ONH) tissues, and for monitoring many ocular^1,2^ and neuro-ocular pathologies^3^. However, despite several advancements in OCT technology^4^, the quality of B-scans is still hampered by speckle noise^5–11^, low signal strength^12^, blink^12,13^ and motion artefacts^12,14^.

Specifically, the granular pattern of speckle noise deteriorates the image contrast, making it difficult to resolve small and low-intensity structures (e.g., sub-retinal layers)^5–7^, thus affecting the clinical interpretation of OCT data. Also, poor image contrast can lead to automated segmentation errors^14–16^, and incorrect tissue thickness estimation^17^, potentially affecting clinical decisions. For instance, segmentation errors for the retinal nerve fiber layer (RNFL) thickness can lead to over/under estimation of glaucoma^18^.

Currently, there exist many hardware^19–27^ and software schemes^27–29^ to denoise OCT B-scans. Hardware approaches offer robust noise suppression through frequency compounding^24–27^ and multi-frame averaging (spatial compounding)^19–23^. While multi-frame averaging techniques have shown to enhance image quality and presentation^28,29^, they are sensitive to registration errors^29^, and require longer scanning times^30^. Moreover, elderly patients often face discomfort and strain^31^, when they remain fixated for long durations^31,32^. Software techniques, on the other hand, attempt to denoise through numerical algorithms^5–11^ or filtering techniques^33–35^. However, registration errors^36^, computational complexity^5,37–39^, and sensitivity to choice of parameters^40^ limit their usage in the clinic.

While deep learning has shown promising segmentation^41–44^, classification^45–47^, and denoising^48–50^ applications in the field of medical imaging for modalities such as magnetic resonance imaging (MRI), its application to OCT imaging is still in its infancy^51–62^. Although recent deep learning studies have shown successful segmentation^51–59^ and classification applications^60–62^ in OCT imaging, to the best of our knowledge no study exists yet to assess the success of denoising OCT B-scans.

In this study, we propose a deep learning approach to denoise OCT B-scans. We aimed to obtain multi-frame quality B-scans (i.e. signal-averaged) from single-frame (without signal averaging) B-scans of the ONH. We hope to offer a denoising framework to obtain superior quality B-scans, with reduced scanning duration and minimal patient discomfort.

## Results

When trained on 23,280 pairs (2,328 B-scans after extensive data augmentation resulted in 23,280 B-scans) of ‘clean’ (multi-frame) and their corresponding ‘noisy’ B-scans (clean B-scans + Gaussian noise), our deep learning network was able to successfully denoise the unseen single-frame B-scans. An independent test set of 1,552 single-frame B-scans was used to evaluate the denoising performance of the proposed network qualitatively and quantitatively.

### Denoising performance – qualitative analysis

**Figure 1:**
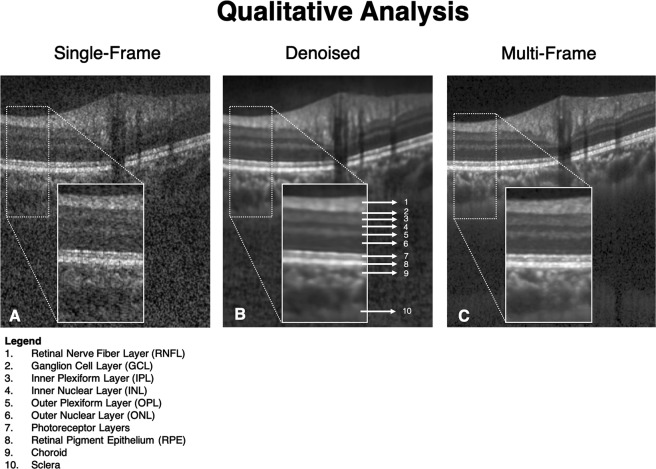
Single-frame (**A**), denoised (**B**), and multi-frame (**C**) B-scans for a healthy subject are shown. The denoised B-scan can be observed to be qualitatively similar to its corresponding multi-frame B-scan. Specifically, the visibility of the retinal layers, and choroid, and lamina cribrosa were prominently improved. Sharp and clear boundaries were also obtained for retinal layers, and the choroid-scleral interface.

**Figure 2:**
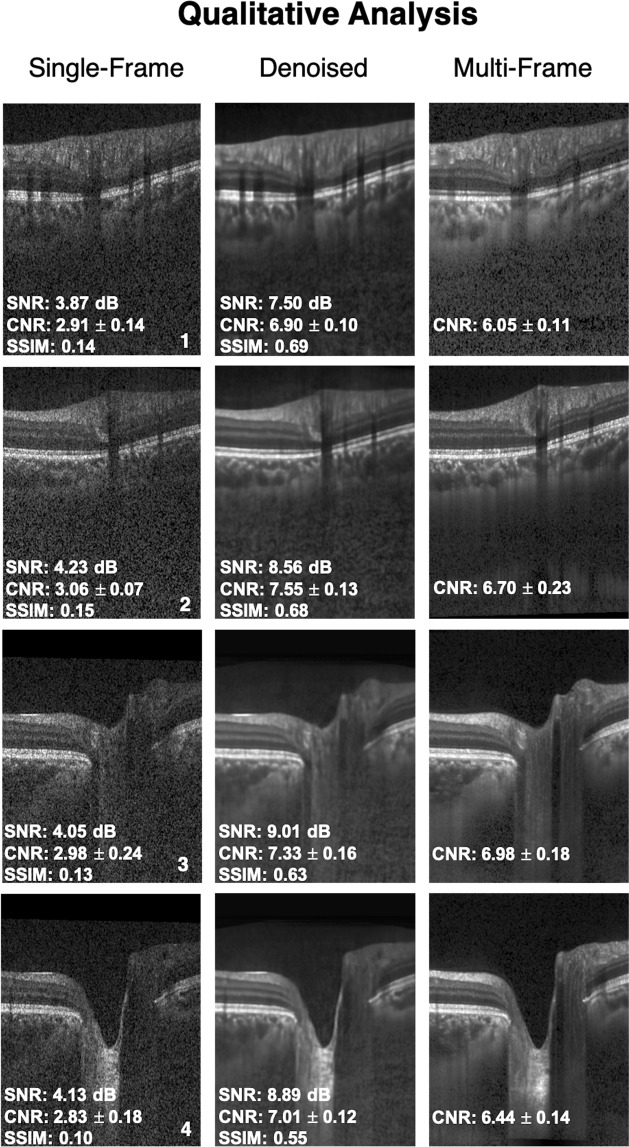
Single-frame, denoised and multi-frame B-scans for four healthy subjects (1–4) are shown. The signal to noise ratio (SNR), contrast to noise ratio (CNR; mean of all tissues) and the structural similarity index (SSIM) for the respective B-scans are shown as well. In all cases, the denoised B-scans (2^nd^ column) were consistently similar (qualitatively) to their corresponding multi-frame B-scans (3^rd^ column).

The single-frame, denoised and multi-frame B-scan for a healthy subject can be found in Fig. 1. In all the cases, the denoised B-scans were qualitatively similar to their corresponding multi-frame B-scans (Fig. 2). Specifically, in all the denoised B-scans, we observed no deep learning induced image artifacts, and the overall visibility of all the ONH tissues were prominently enhanced (Fig. 2; 2^nd^ column).

### Denoising performance – quantitative analysis

When evaluated on the independent test set of 1,552 B-scans, on average, we observed a two-fold increase in SNR upon denoising. Specifically, the mean SNR for the unseen single-frame/denoised B-scans were: 4.02 ± 0.68 dB/8.14 ± 1.03 dB, respectively, when computed against their respective multi-frame B-scans. The two-fold reduction in the noise levels resulted in the enhanced overall visibility of the ONH tissues.

In all cases, the multi-frame B-scans always offered a higher CNR compared to their corresponding single-frame B-scans. Further, the denoised B-scans consistently offered a higher CNR compared to the single-frame B-scans, for all tissues (Table 1). Specifically, the mean CNR (mean of all tissues) increased from 3.50 ± 0.56 (single-frame) to 7.63 ± 1.81 (denoised). For each tissue, mean CNR in a single-frame, denoised and multi-frame B-scan can be found in Table 1. The increased CNR values for each ONH tissue in the denoised images implied sharper and improved visibility of the tissue boundaries.

**Table 1:** Mean CNR for all ONH tissues computed for the single-frame, denoised and multi-frame B-scans.

Tissue	Single-frame	Denoised	Multi-frame
RNFL	2.97 ± 0.42	7.28 ± 0.63	5.18 ± 0.76
GCL + IPL	3.83 ± 0.43	12.09 ± 4.22	11.62 ± 1.85
All other retinal layers	2.71 ± 0.33	5.61 ± 1.46	4.62 ± 0.86
RPE	5.62 ± 0.72	9.25 ± 2.25	8.10 ± 1.44
Choroid	2.99 ± 0.43	5.99 ± 0.45	5.75 ± 0.63
Sclera	2.42 ± 0.39	6.40 ± 1.68	6.00 ± 0.96
LC	4.02 ± 1.23	6.81 ± 1.99	6.46 ± 1.81

Finally, on average, our denoising approach offered a five-fold increase in MSSIM. Specifically, the mean MSSIM for the single-frame/denoised B-scans were: 0.13 ± 0.02/0.65 ± 0.03, when computed against their respective multi-frame B-scans. Thus, the denoised B-scans were five-times structural more similar (compared to the single-frame B-scans) to the multi-frame B-scans.

### Denoising performance – effect of data augmentation

**Figure 3:**
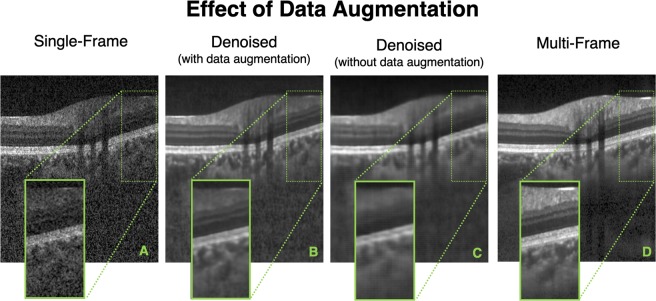
Single-frame (**A**), denoised (with data augmentation) (**B**), denoised (without data augmentation) (**C**), and multi-frame (**D**) B-scans for a healthy subject are shown. The denoised B-scan (**B**) obtained from a network trained with data augmentation can be observed to be qualitatively similar to its corresponding multi-frame B-scan (**D**). However, when trained with limited training data (without data augmentation), although the network is able to reduce the speckle noise primarily, the denoised B-scan is over-smoothened (**C**) with smudged and unclear tissue boundaries.

When trained without data augmentation, in all the test cases, while the network was able to reduce the speckle noise primarily, the denoised B-scans appeared to be over-smoothened (blurred). Specifically, the tissue boundaries (especially for the retinal layers) appeared smudged and unclear (Fig. 3).

While the network trained with the baseline dataset (without data augmentation) offered a slightly higher mean SNR of 10.11 ± 2.13 dB, (vs. with data augmentation: 8.14 ± 1.03 dB), the higher SNR can be attributed to the over-smoothened (blur) denoised B-scans, and not the improved image quality and reliability.

Further, we would like to clarify that we were unable to obtain reliable CNR values for the individual ONH tissues in all the denoised images (network trained without data augmentation) due to smudged and unclear tissue boundaries.

Finally, while the network trained with the baseline dataset resulted in denoised B-scans with nearly two-fold increased mean MSSIM (0.25 ± 0.07; ‘noisy’ B-scans mean MSSIM: 0.13 ± 0.02), the use of data augmentation helped retrieve structural information (mean MSSIM: 0.65 ± 0.03; five-fold increase compared to ‘noisy’ B-scans).

Overall, we observed that a 10-fold increase in the dataset size through extensive data augmentation did improve the overall quality and reliability of the denoised B-scans.

### Denoising performance: clinical reliability

For all the three structural parameters, there were no significant (p > 0.05) differences (means) in the measurements when obtained from the denoised or the multi-frame radial B-scans.

The percentage errors (denoised vs. multi-frame radial B-scans; mean ± standard deviation) in the measurements of the p-RNFLT, the p-GCCT, and the p-CT were 3.07 ± 0.75%, 2.95 ± 1.02%, and 3.90 ± 2.85% respectively.

## Discussion

In this study, we present a custom deep learning approach to denoise single-frame OCT B-scans of the ONH. When trained with the ‘clean’ (multi-frame) and the corresponding ‘noisy’ B-scans, our network denoised unseen single-frame B-scans. The proposed network leveraged on the inherent advantages of U-Net, residual learning, and dilated convolutions^58^. Further, the multi-scale hierarchical feature extraction^63^ pathway helped the network recover ONH tissue boundaries degraded by speckle noise. Having successfully trained, tested and validated our network on 1,552 single-frame OCT B-scans of the ONH, we observed a consistently higher SNR and CNR for all ONH tissues, and a consistent five-fold increase in MSSIM in all the denoised B-scans. Thus, we may be able to offer a robust deep learning framework to obtain superior quality OCT B-scans with reduced scanning duration and minimal patient discomfort.

Using the proposed network, we obtained denoised B-scans that were qualitatively similar to their corresponding multi-frame B-scans (Figs 1 and 2), owing to the reduction in noise levels. The mean SNR for the denoised B-scans was 8.14 ± 1.03 dB, a two-fold improvement (reduction in noise level) from 4.02 ± 0.68 that was obtained for the single-frame B-scans. Given the significance of the neural (retinal layers)^64–68^ and connective tissues (sclera and LC)^69–73^, in ocular pathologies such as glaucoma^2^, and age-related macular degeneration^74^, their enhanced visibility is critical in a clinical setting. Furthermore, reduced noise levels would likely increase the robustness of aligning/registration algorithms used to monitor structural changes over time^17^. This is crucial for the management of multiple ocular pathologies^75,76^.

In denoised B-scans (vs single-frame B-scans), we consistently observed higher CNRs. Our approach enhanced the visibility of small (e.g. RPE and photoreceptors) and low-intensity tissues (e.g. GCL and IPL; Fig. 2: 2^nd^ column). For all tissues, the mean CNR increased from 3.50 ± 0.56 (single-frame) to 7.63 ± 1.81 (denoised). Since existing automated segmentation algorithms rely on high contrast, we believe that our approach could potentially reduce the likelihood of segmentation errors that are relatively common in commercial algorithms^14–16,77^. For instance, the incorrect segmentation of the RNFL can lead to inaccurate thickness measurements, leading to under-/over- estimation of the glaucomatous damage^18^. By using the denoising framework as a precursor to automated segmentation/thickness measurement, we could increase the reliability^78^ of such clinical tools.

Upon denoising, we observed a five-fold increase in MSSIM (single-frame/denoised: 0.13 ± 0.02/0.65 ± 0.03), when validated against the multi-frame B-scans. The preservation of features and structural information plays an important role in accurately measuring cellular level disruption to determine retinal pathology. For instance, the measurement of the ellipsoid zone (EZ) disruption^79^ provides an insight into the photoreceptor structure, that is significant in pathologies such as diabetic retinopathy^80^, macular hole^81^, macular degeneration^82^, and ocular trauma^83^. Existing multi-frame averaging techniques^29^ significantly enhance and preserve the integrity of the structural information by supressing speckle noise^30,39–41^. However, they are limited by a major clinical challenge: the inability of the patients to remain fixated for long scanning times^31,32^, and the resultant discomfort^31^.

In this study, we are proposing a methodology to significantly reduce scanning time while enhancing OCT signal quality. In our healthy subjects, it took on average 3.5 min to capture a ‘clean’ (multi frame) volume, and 25 s for a ‘noisy’ (single frame) volume. Since we can denoise a single B-scan in 20 ms (or 2 s for a volume of 97 B-scans), this means that we can theoretically generate a denoised OCT volume in about 27 seconds (=time of acquisition of the ‘noisy’ volume [25 s] + denoising processing [2 s]). Thus, we may be able to drastically reduce the scanning duration by more than 7 folds, while maintaining superior image quality.

Besides speckle noise, patient dependent factors such as cataract^84–87^ and/or lack of tear film in dry eyes can significantly diminish OCT scan quality^12,84–88^. While lubricating eye drops and frequent blinking can instantly improve image quality for patients with corneal drying^88,89^, the detrimental effects of cataract on OCT image quality might be reduced only if cataract surgery is performed^12,84,85^. Moreover, pupillary dilation may be needed especially in subjects with small pupil sizes to obtain acceptable quality B-scans^12,90^, which is highly crucial in the monitoring of glaucoma^90^. Pupillary dilation is also time consuming and may cause patient discomfort^91^. It is plausible that the proposed framework, when extended, could be a solution to the afore-mentioned factors that limit image quality, avoiding the need for any additional clinical procedure.

In this study, several limitations warrant further discussion. First, the proposed network was trained and tested only on B-scans from one device (Spectralis). Every commercial OCT device has its own proprietary algorithm to pre-process the raw OCT data, potentially presenting a noise distribution different from what our network was trained with. Hence, we are unsure of our network’s performance on other devices. Nevertheless, we offer a proof of concept which could be validated by other groups on multiple commercial OCT devices.

Second, we were unable to train our network with a speckle noise model representative of the Spectralis device. Such a model is currently not provided by the manufacturer and would be extremely hard to reverse-engineer because information about all pre- and post-processing done to the OCT signal is also not provided. While there exist a number of OCT denoising studies that assume a Rayleigh^8^/Generalized Gamma distribution to describe speckle noise^39^, we observed that they were ill-suited for our network. From our experiments, the best denoising performance was obtained when our network was trained with a simple Gaussian noise model (μ = 0, σ = 1). It is possible that a thorough understanding of the raw noise distribution prior to the custom pre-processing on the OCT device could improve the performance of our network. We aim to test this hypothesis with a custom-built OCT system in our future works.

Third, while we have discussed the need for reliable clinical information from poor quality OCT scans, that could be critical for the diagnosis and management of ocular pathology (e.g., glaucoma), we have yet to test the networks’ performance on pathological B-scans.

Fourth, we observed that the CNR was higher for the denoised B-scans than for the corresponding multi-frame B-scans. This could be attributed to over-smoothening (or blurring) of tissue textures that was consistently present in the denoised B-scans. We are currently exploring other deep learning techniques to improve the B-scan sharpness that is lost during denoising.

Fifth, we were unable to provide further validation of our algorithm by comparing our outputs to histology data. Such a validation would be extremely difficult, as one would need to first image a human ONH with OCT, process with histology, and register both datasets. Furthermore, while we believe our algorithm is able to restore tissue texture accurately (when comparing denoised B-scans with multi-frame B-scans), an exact validation of our approach is not possible. Long fixation times in obtaining the multi-frame B-scans lead to subtle motion artifacts (eye movements caused by microsaccades or unstable fixation)^92^, displaced optic disc center^93^, and axial misalignment^12^, causing minor registration errors between the single-frame and multi-frame B-scans, thus preventing an exact comparison between the denoised B-scans and the multi-frame B-scans.

Finally, although we observed no significant differences in the measurements of the p-RNFLT, p-GCCT and the p-CT when measured on the denoised or the multi-frame B-scans, it must be noted that the measurements were obtained on a testing cohort of limited (8 subjects) and healthy subjects only. Further studies on larger and diverse (presence of pathology) cohorts are necessary to assert and robustly validate the clinical relevance of the proposed technique.

In conclusion, we have developed a custom deep learning approach to denoise single-frame OCT B-scans. With the proposed network, we were able to denoise a single-frame OCT B-scan in under 20 ms. We hope that the proposed framework could resolve the current trade-off in obtaining reliable and superior quality scans, with reduced scanning times and minimal patient discomfort. Finally, we believe that our approach may be helpful for low-cost OCT devices, whose noisy B-scans may be enhanced by artificial intelligence (as opposed to expensive hardware) to the same quality as in current commercial devices.

## Methods

### Patient recruitment

A total of 20 healthy subjects were recruited at the Singapore National Eye Centre. All subjects gave written informed consent. This study was approved by the institutional review board of the SingHealth Centralized Institutional Review Board and adhered to the tenets of the Declaration of Helsinki. The inclusion criteria for healthy subjects were: an intraocular pressure (IOP) less than 21 mmHg, and healthy optic nerves with a vertical cup-disc ratio (VCDR) less than or equal to 0.5.

### Optical coherence tomography imaging

The subjects were seated and imaged under dark room conditions by a single operator (TAT). A spectral-domain OCT (Spectralis, Heidelberg Engineering, Heidelberg, Germany) was used to image both eyes of each subject. Each OCT volume consisted of 97 horizontal B-scans (32-μm distance between B-scans; 384 A-scans per B-scan), covering a rectangular area of 15° × 10° centered on the ONH. For each eye, single-frame (without signal averaging), and multi-frame (75x signal averaging) volume scans were obtained. Enhanced depth imaging (EDI)^94^ and eye tracking^92,95^ modalities were used during the acquisition. From all the subjects, we obtained a total of 3,880 B-scans for each type of scan (single-frame or multi-frame).

### Volume registration

The multi-frame volumes were reoriented to align with the single-frame volumes through rigid translation/rotation transformations using 3D software (Amira, version 5.6; FEI). This registration was performed using a voxel-based algorithm that maximized mutual information between two volumes^96^. Registration was essential to quantitatively validate the corresponding regions between the denoised and multi-frame B-scans. Note that Spectralis follow-up mode was not used in this study. Although the follow-up mode allows a new scanning of the same area by identifying previous scan locations, in many cases, it can distort B-scans and thus provide unrealistic tissue structures in the new scan.

### Deep learning based denoising

In this study, we developed a fully-convolutional neural network, inspired by our earlier DRUNET architecture^58^ to denoise single-frame OCT B-scans of the ONH. It leverages on the inherent advantages of U-Net^97^, residual learning^98^, dilated convolutions^99^, and multi-scale hierarchical feature extraction^63^ to obtain multi-frame quality B-scans. Briefly, the U-Net and its skip connections helped the network learn both the local (tissue texture) and contextual information (spatial arrangement of tissues). The contextual information was further exploited using dilated convolution filters. Residual connections improved the flow of the gradient information through the network, and multi-scale hierarchical feature extraction helped restore tissue boundaries in the B-scans.

#### Network architecture

The proposed network consisted of two types of feature extraction units: **(1)** standard block; and **(2)** residual block. While each block consisted of two subsequent dilated convolutional layers (64 filters; size = 3 × 3) for feature extraction, the residual block consisted of an additional 3 × 3 convolutional layer (identity connection)^98^ that improved the flow of the gradient information throughout the depth of the network. The use of dilated convolution filters^99^ helped the network better understand the contextual information (i.e., the spatial arrangement of tissues), that is crucial for obtaining reliable tissue boundaries.

**Figure 4:**
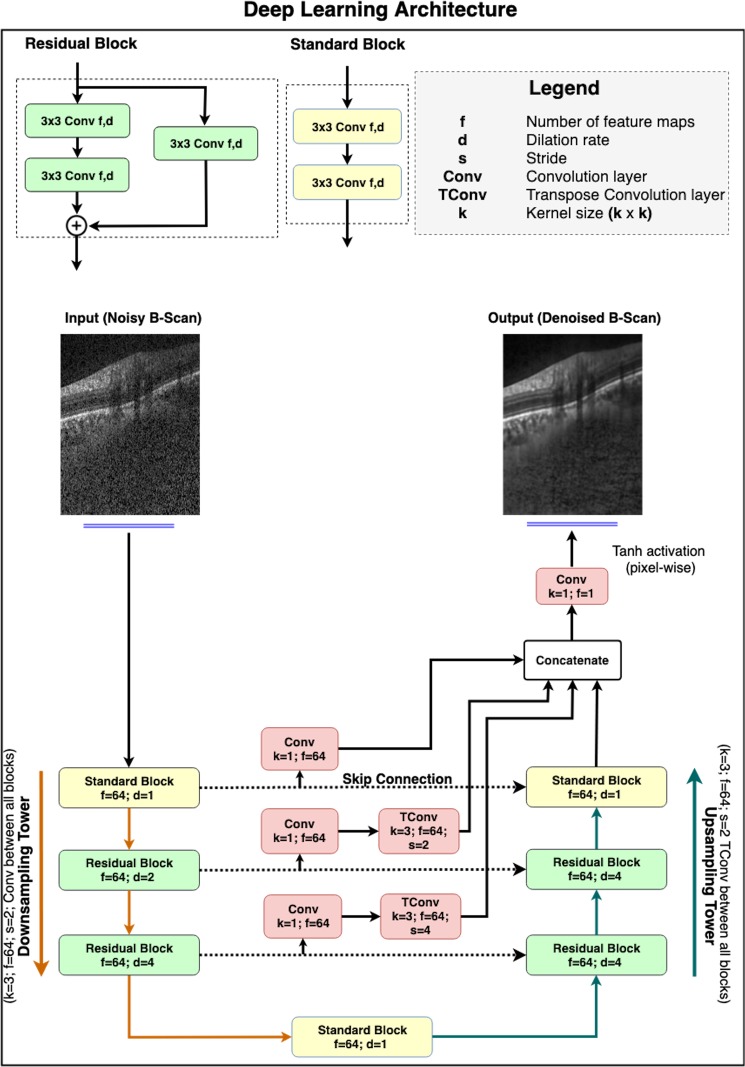
The architecture comprised of two towers: (1) A downsampling tower – to capture the contextual information (i.e., spatial arrangement of the tissues), and (2) an upsampling tower – to capture the local information (i.e., tissue texture). Each tower consisted of two blocks: (1) a standard block, and (2) a residual block. The latent space was implemented as a standard block. The multi-scale hierarchical feature extraction unit helped better recover tissue edges eroded by speckle noise. The network consisted of 900 k trainable parameters.

Inspired by the U-Net^97^, the network was composed of a downsampling and an upsampling tower, connected via skip-connections (Fig. 4). By sequentially halving the dimensionality of the B-scan after each feature extraction unit, the downsampling tower extracted the contextual information (i.e., the spatial arrangement of tissues). On the other hand, in the process of sequentially restoring the B-scan to its original dimensions, the upsampling tower extracted local information (i.e., tissue texture). Skip connections between both towers helped the network learn the extracted contextual and local information jointly.

In the downsampling tower, an input B-scan (size: 496 × 384) was first passed on to a standard block (dilation rate: 1) followed by two residual blocks (dilation rate: 2 and 4, respectively). A convolution layer (64 filters; size = 3 × 3; stride = 2) after every block sequentially halved the dimensionality of the B-scan.

A standard block (dilation rate: 1) was then used to transfer the contextual feature maps from the downsampling to the upsampling tower.

The upsampling tower consisted of two residual blocks (dilation rate: 4) and a standard block (dilation rate: 1). After each block, a transpose convolution layer (64 filters; size = 3 × 3; stride = 2) was used to restore the B-scan sequentially to its original dimension.

The use of multi-scale hierarchical feature extraction^63^ improved the recovery of tissue boundaries eroded by speckle noise in the single-frame B-scans. It was implemented by passing the feature maps at each downsampling level through a convolution layer (64 filters; size = 1 × 1), followed by a transpose convolution layer (64 filters; size = 3 × 3) to restore the original B-scan resolution. The restored maps were then concatenated with the output feature maps from the upsampling tower.

Finally, the concatenated feature maps were fed to the output convolution layer (1 filter; size = 1 × 1), followed by pixel-wise hyperbolic tangent (tanh) activation to produce a denoised B-scan.

In both towers, all layers except the last output layer, were activated by an exponential linear unit (ELU)^100^ function. In addition, in each residual block, the feature maps were batch normalized^101^ and ELU activated before addition.

The proposed network comprised of 900,000 training parameters. The network was trained end-to-end using the Adam optimizer^102^, and we used the mean absolute error as loss function. We trained and tested the proposed network on an NVIDIA GTX 1080 founders edition GPU with CUDA v8.0 and cuDNN v5.1 acceleration. With the given hardware configuration, each single-frame B-scan was denoised under 20 ms.

#### Training and testing of the network

**Figure 5:**
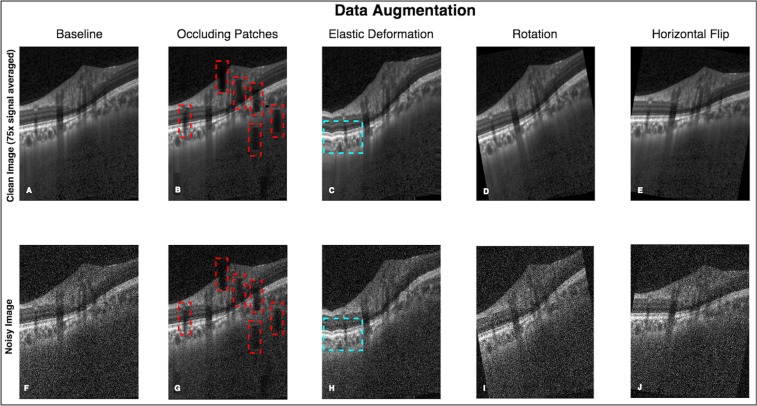
An exhaustive offline data augmentation was done to circumvent the scarcity of training data. (**A–E**) represent the original and the data augmented ‘clean’ B-scans (multi-frame). (**F–J**) Represent the same for the corresponding ‘noisy’ B-scans. The occluding patches (**B,G**; red boxes) were added to make the network robust in the presence of blood vessel shadows. Elastic deformations (**C,H**; cyan boxes) were used to make the network invariant to atypical morphologies^104^. A total of 23,280 B-scans of each type (clean/noisy) were generated from 2,328 baseline B-scans.

From the dataset of 3,880 B-scans, 2,328 of them (from both eyes of 12 subjects) were used as a part of the training dataset. The training set consisted of ‘clean’ B-scans and their corresponding ‘noisy’ versions. The ‘clean’ B-scans were simply the multi-frame (75x signal averaging) B-scans. The ‘noisy’ B-scans were generated by adding Gaussian noise (μ = 0, σ = 1) to the respective ‘clean’ B-scans (Fig. 5).

The testing set consisted of 1,552 single-frame B-scans (from both eyes of 8 subjects) to be denoised. We ensured that the scans from the same subject weren’t used in both training and testing sets.

#### Data augmentation

An exhaustive offline data augmentation was done to circumvent the scarcity of training data. We used elastic deformations^58,103^, rotations (clockwise and anti-clockwise; 10°), occluding patches^58^, and horizontal flipping for both ‘clean’ and ‘noisy’ B-scans. Briefly, elastic deformations were used to produce the combined effects of shearing and stretching in an attempt to make the network invariant to atypical morphologies (as seen in glaucoma^104^). Ten occluding patches of size 60 × 20 pixels were added at random locations to non-linearly reduce (pixel intensities multiplied by a random factor between 0.2 and 0.8) the visibility of the ONH tissues. This was done to make the network invariant to blood vessel shadows that are common in OCT B-scans^105^. Note that a full description of our data augmentation approach can be found in our previous paper^58^.

Using data augmentation, we were able to generate a total of 23,280 ‘clean’ and 23,280 corresponding ‘noisy’ B-scans that were added to the training dataset. An example of data augmentation performed on a single ‘clean’ and corresponding ‘noisy’ B-scan is shown in Fig. 5.

### Denoising performance – qualitative analysis

All denoised single-frame B-scans were manually reviewed by expert observers (S.K.D. & G.S.) and qualitatively compared against their corresponding multi-frame B-scans to assess the following: 1) presence of deep learning induced image artifacts in the denoised B-scans; and 2) overall visibility of the ONH tissues.

### Denoising performance – quantitative analysis

The following image quality metrics were used to assess the denoising performance of the proposed algorithm: **(1)** signal to noise ratio (SNR); **(2)** contrast to noise ratio (CNR); and **(3)** mean structural similarity index measure (MSSIM)^106^. These metrics were computed for the single-frame, multi-frame, and denoised B-scans (all from the independent testing set; 1,552 B-scans of each type).

The SNR (expressed in dB) was a measure of signal strength relative to noise. It was defined as:

1 $$SNR=-\,10\ast lo{g}_{10}(\frac{\Vert {f}_{o}-{\tilde{f}}^{2}\Vert }{\Vert {{f}_{o}}^{2}\Vert })$$

where ${f}_{o}$ is the pixel-intensity values of the ‘clean’ (multi-frame) B-scan, and $\tilde{f}$is the pixel-intensity values of the B-scan (either the ‘noisy’ [single-frame] or the denoised B-scan) to be compared with ${f}_{o}$. A high *SNR* value indicates low noise in the given B-scan with respect to the ‘clean’ B-scan.

The CNR was a measure of contrast difference between different tissue layers. It was defined as:

2 $$CN{R}_{i}=\frac{|{\mu }_{r}-{\mu }_{b}|}{\sqrt{0.5({\sigma }_{r}^{2}+{\sigma }_{b}^{2})}}$$

where *μ*_*r*_ and ${\sigma }_{r}^{2}$ denoted the mean and variance of pixel intensity for a chosen ROI within the tissue ‘*i*’ in a given B-scan, while *μ*_*b*_ and ${\sigma }_{b}^{2}$ represented the same for the background ROI. The background ROI was chosen as a 20 × 384 (in pixels) region at the top of the image (within the vitreous). A high CNR value suggested enhanced visibility of the given tissue.

The CNR was computed for the following tissues: **(1)** RNFL; **(2)** ganglion cell layer + inner plexiform layer (GCL + IPL); **(3)** all other retinal layers; **(4)** retinal pigment epithelium (RPE); **(5)** peripapillary choroid; **(6)** peripapillary sclera; and **(7)** lamina cribrosa (LC). Note that the CNR was computed only in the visible portions of the peripapillary sclera and LC. For each tissue, the CNR was computed as the mean of twenty-five ROIs (8 × 8 pixels each) in a given B-scan. All the ROIs were manually chosen in each tissue by an expert observer (G.S.) using a custom MATLAB (R2015a, MathWorks Inc., Natick, MA) graphical user interface.

The structural similarity index measure (SSIM)^106^ was computed to assess the changes in tissue structures (i.e., edges) between the single-frame/denoised B-scans and the corresponding multi-frame B-scans (ground-truth). The SSIM was defined between −1 and +1, where −1 represented ‘no similarity’, and +1 ‘perfect similarity’. It was defined as:

3 $$SSIM(x,y)=\frac{(2{\mu }_{x}{\mu }_{y}+{\complement }_{1})(2{\sigma }_{xy}+{\complement }_{2})}{({\mu }_{x}^{2}+{\mu }_{y}^{2}+{\complement }_{1})({\sigma }_{x}^{2}+{\sigma }_{y}^{2}+{\complement }_{2})}$$

where *x* and *y* represented the denoised and multi-frame B-scan respectively; ${\mu }_{x}$, ${\sigma }_{x}^{2}$ denoted the mean intensity and standard deviation of the chosen ROI in B-scan x, while ${\mu }_{y}$, $\,{\sigma }_{y}^{2}$ represented the same for B-scan y; ${\sigma }_{xy}$ represented the cross-covariance of the ROIs in B-scans *x* and *y*. C_1_ and C_2_ (constants to stabilize the division) were chosen as 6.50 and 58.52, as recommended in a previous study^106^.

The MSSIM was computed as the mean of SSIM from ROIs (8 × 8 pixels each) across a B-scan (stride = 1; scanned horizontally). It was defined as:

4 $$MSSIM(X,Y)=\frac{1}{M}\mathop{\sum }\limits_{k=1}^{M}SSIM({x}_{k},{y}_{k})$$

Note that the SNR, and MSSIM were computed for an entire B-scan, as opposed to the CNR that was computed for individual tissues.

### Denoising performance: effect of data augmentation

In an attempt to understand the significance of data augmentation, the entire process of training and testing was performed on two datasets: (1) baseline dataset (without data augmentation; 2,328 pairs of ‘clean’ and corresponding ‘noisy’ B-scans); and (2) data augmented dataset (23,280 pairs of ‘clean’ and corresponding ‘noisy’ B-scans).

### Denoising performance: clinical reliability

The clinical reliability of the denoised B-scans was assessed by comparing the measurements of 3 clinically relevant ONH structural parameters obtained from the denoised and its corresponding multi-frame radial B-scans.

For each of the eight subjects in the testing set, we obtained 12 radial B-scans passing through the center of the Bruch’s membrane opening (BMO) from both the denoised volumes (single-frame volumes denoised using the proposed network) and their corresponding multi-frame volumes. A total of 192 pairs (8 subjects; both eyes; 12 B-scans per volume) of radial B-scans (denoised and its corresponding multi-frame B-scans) were obtained.

In all the radial B-scans, the peripapillary retinal nerve fiber layer thickness (p-RNFLT), the peripapillary ganglion cell complex layer thickness (ganglion cell layer + inner plexiform layer; p-GCCT), and the peripapillary choroidal thickness (p-CT) were manually measured by an expert observer (S.K.D.) using ImageJ^107^.

In each radial B-scan, the BMO points were defined as the extreme-tips of the RPE. The BMO reference line was obtained by joining the BMO points.

The p-RNFLT was computed as the distance between the inner limiting membrane and the posterior RNFL boundary taken at 1.7 mm on either side from the center of the BMO reference line.

The p-GCCT was computed as the distance between the posterior RNFL boundary and the posterior inner plexiform layer boundary taken at 1.7 mm on either side from the center of the BMO reference line.

The p-CT was computed as the distance between the posterior RPE boundary and the choroid-sclera interface taken at 1.7 mm on either side from the center of the BMO reference line.

All the three structural parameters were calculated as the average of the measurements taken on either side of the BMO.

For each structural parameter, paired t-test were used to assess the difference (means) in measurements when obtained from the denoised and their corresponding multi-frame radial B-scans.

Finally, we also computed the percentage error (mean) in measurements (between denoised and multi-frame radial B-scans) for each parameter.

## Data Availability

The dataset used for the training, validation, and testing of the proposed deep learning network was obtained from the Singapore National Eye Center and transferred to the National University of Singapore in a de-identified format. The study was approved by the SingHealth Centralized Institutional Review. Due to regulations, SingHealth does not authorize the dataset to be shared publicly.

## Code Availability

The authors wish to license the code to another party and thus are unable to release the entire codebase publicly at this stage. However, all the experiments, network descriptions, and data augmentations are described in sufficient details to enable independent replication with non-proprietary libraries.
